# Medicinal plants for renal injury prevention

**DOI:** 10.12861/jrip.2013.21

**Published:** 2013-06-01

**Authors:** Mahmoud Rafieian-kopaei

**Affiliations:** ^1^Medical Plants Research Center, Shahrekord University of Medical Sciences, Shahrekord, Iran

**Keywords:** Nephropathy, Kidney, Nephroprotection, Antioxidants

## Abstract

It has been estimated that about 20% of men and 25% of women between the ages of 65 and 74 have some degrees of chronic kidney. This complication is attributed to oxidative stress. Oxidative stress is an important factor contributing to kidney damage by increasing production of oxidants, particularly insufficiency of endogenous antioxidant defense system. Medicinal plants antioxidants are able to ameliorate oxidative induced kidney damage by reduction of lipid peroxidation and enhancement of scavenging ability of antioxidant defense system. Supplementation of medicinal plants antioxidants might be considered important remedies to abrogate pathology of oxidative stress induced kidney damage, however, single antioxidants do not act the same and might not be beneficial.

**Implication for health policy/practice/research/medical education:**

Oxidative stress is an important factor contributing to kidney damage by increasing production of oxidants, particularly insufficiency of endogenous antioxidant defense system. Medicinal plants antioxidants have been shown to ameliorate oxidative induced kidney damage by reduction of lipid peroxidation and enhancement of scavenging ability of antioxidant defense system. Supplementation of medicinal plants antioxidants might be considered important remedies to abrogate pathology of oxidative stress induced kidney damage, however, single antioxidants do not act the same and might not be beneficial.

## Introduction

More than one fifth of people over ages of 65 years have some degrees of chronic kidney disease (CKD) (1). This complication is attributed to oxidative stress. Oxidative stress is an important factor contributing to kidney damage by increasing production of oxidants, particularly insufficiency of endogenous antioxidant defense system (1).

Medicinal plants antioxidants are able to ameliorate oxidative induced kidney damage by reduction of lipid peroxidation and enhancement of scavenging ability of antioxidant defense system. Supplementation of medicinal plants antioxidants might be considered important remedies to abrogate pathology of oxidative stress induced kidney damage, however, single antioxidants do not act the same and might not be beneficial. This paper reviews the effects of antioxidants and tries to help choosing suitable antioxidant for pathologic kidney injury. It has been estimated that about 20% of men and 25% of women between the ages of 65 and 74 have some degrees of chronic kidney. CKD is more common in south Asian people especially those from Pakistan, Bangladesh, India and Sri Lanka as well as black people. The reasons for this include higher rates of high blood pressure in African people and higher diabetes in south Asia. These are two diseases attributed to oxidative stress (1). Although there are some ways to slow down or halt the progression of CKD, however, there is no cure for this problem. Patients with CKD are known to have a higher risk of stroke or heart attack due to changes that may occur to their circulation (1,2). CKD may cause kidney failure or end-stage kidney disease, in which kidney may stop working. The main way to inhibit CKD is management of the existing conditions, such as high blood pressure and diabetes. Changing the lifestyle, avoiding alcohol drinking and healthy diets are important factors to reduce CKD. However, most of these and other conditions resulting kidney injury are associated with oxidative stress (OS). Hence, OS is one of the most important factors associated with kidney injury (1-3). This review emphasizes the effects of antioxidants from medicinal plant on pathologies of kidney injury.

### Antioxidant systems

Oxidative stress is the imbalance between the rate of production and removal of produced oxidants. In other word it is an increase in reactive oxygen species (ROS) and reactive nitrogen species (RNS) and/or decrease in endogenous/exogenous antioxidants. It is the causative factor of a wide variety of diseases such as neurodegenerative diseases, diabetes, atherosclerosis, ischemia, and kidney disease (4,5).The most important ROS molecules are hydroxyl radical (•OH), superoxide anion (O2•-), hydrogen peroxide (H2O2) and hypochlorous acid (HOCl). ROS molecules are highly reactive and are cable of becoming toxic to macromolecules, such as DNA, proteins and lipids. Oxidant molecules are produced in both endogenous and exogenous sources. Endogenous sources of ROS are consisted of oxidant enzymes, auto-oxidation reaction mitochondrial electron transport chain, and phagocytes (6,7). Exogenous ROS molecules consisted of xenobiotics, cigarettes and alcohols, radiations, chlorinated compounds and so on (8). RNS molecules are by-products of nitric oxide (•NO). The same as ROS, they play a key role in maintaining various physiological functions, however, at high levels they can contribute to a number of pathological conditions. RNS molecules are consisted of peroxynitrite (ONOO-), nitrite (NO2-), and nitrate (NO3) (8).The body has a defense system (antioxidant system) which combats the ROS/RNS-caused cellular damage. Antioxidants are molecules which are able to inhibit the oxidation of substrates (9). Depends on their mechanism of action, antioxidants can be divided into two types of breaking or preventive antioxidants (10). Preventive antioxidants are able to quench singlet oxygen and reduce the rate of chain initiation by deactivating metals and decreasing hydroperoxides. Chain breaking antioxidants are able to donate or receive an electron from a radical with the formation of stable byproducts such as β-carotene, ascorbic acid, uric acid α-tocopherol (6,9).

### Medicinal plants antioxidants

Medicinal plants are considered as healthy sources for the prevention of various oxidative stress-related diseases (11). Currently there have been increasing interests in the advantage of medicinal plants. They possess a lot of phytochemical constituents with antioxidant activities including phenolic compounds and carotenoids (12,13) which have antioxidant properties such as chain breaking antioxidants. Intake of carotenoids has shown a significant reduction in the risk of several chronic and degenerative diseases (14). Phenolic compounds are usually found in medicinal plants and food products and mainly consisted of phenolic acids, flavonoids and tannins. These compounds have a wide range of antioxidant activities (15,16).

### Protective effect of antioxidants on kidney damage

Oxidative stress induced kidney damage is associated with increased ROS/RNS production. Moreover, oxidative stress induced kidney damage is significantly reduced by antioxidants (17).

Medicinal plants-derived antioxidants can protect renal damage through reduction of lipid peroxidation (LPO) and increase in endogenous antioxidants. Increased levels and activities of endogenous antioxidants reduce kidney damage. Tocotrienol, a member of vitamin E family, supplementation exhibited the capacity to reduce proximal tubular injury and renal LPO, and increased GSH level and catalase activity. Moreover, it is able to improve the index of NO2 -/NO3 –generation. Tocotrienol can be considered a natural antioxidant supplement protecting the kidney pathology induced by potassium dichromate (18). Ligustrazine, an alkaloid extracted from Ligusticum wallichii with antioxidant activity has been able to protect kidneys from ischemia/reperfusion injuries by elevating SOD activity, decreasing ROS generation and reducing MDA. Troxerutin which is found abundant in cereal grain, tea, coffee and a variety of fruits and vegetables has been shown to elevate antioxidant enzyme activities, including Cu/Zn SOD, GPx and catalase and to reduce MDA level (19). Recent studies have shown that it has the ability to reduce oxidative stress-induced kidney damage (20).As mentioned above, antioxidants usually work by giving electrons to free radicals without turning into electron-scavenging substances themselves. It has been found that people with low intake of vegetables and fruits were at greater risk for development of some diseases compared to others. Although free radicals contribute to kidney injury (21,22), diabetes (23,24), atherosclerosis (22,23), heart disease, nephrotoxicity, hepatotoxicity, cognitive (21-24) and vision loss (25) and a lot of researches, especially laboratory trials, show benefits for antioxidants against these conditions, however, long clinical trials do not uniformly confirm that antioxidant supplements have a substantial impact on these diseases. It seems that the molecules which found naturally in vegetables, fruits, and grains help prevent a variety of conditions such as kidney injury, antioxidants in all conditions do not act the same. The results of the large studies offer little evidence that taking single antioxidants such as vitamin E or vitamin C protect against kidney injury as well as other oxidative stress related diseases (21-24). While the results of the large studies offer little evidence that taking single antioxidants protect against conditions such as kidney injury, the findings about combinations are also complicated and not entirely clear (26).What is clear is that natural whole products such as vegetables and fruits are capable of preventing or curing a variety of chronic diseases such as kidney diseases but single antioxidants or even their combination do not act the same, however, the reason is not clear. Possibly because antioxidants usually act as parts of elaborate networks and therefore, no single antioxidant can do the work of the whole crowd (2). Although it has been evidenced that eating whole vegetables, fruits and grains, which all are rich in antioxidants, provides protection against oxidative stress induced diseases such as kidney injury, however, this does not mean that antioxidants will prevent or fix the problem, especially not when they are taken out of their natural context. It should be noted that although the results of the studies are inconclusive, but most of the studies conducted till now have had limitations due to their relatively short duration and conducting on patients with existing diseases.

## Conclusion

Oxidative stress is an important factor contributing to kidney damage by increasing production of oxidants, particularly insufficiency of endogenous antioxidant defense system. Medicinal plants antioxidants have been shown to ameliorate oxidative induced kidney damage by reduction of lipid peroxidation and enhancement of scavenging ability of antioxidant defense system. Supplementation of medicinal plants antioxidants might be considered important remedies to abrogate pathology of oxidative stress induced kidney damage, however, single antioxidants do not act the same and might not be beneficial.

## Author’s contribution

MRK is the single author of the manuscript.

## Conflict of interests

The author declared no competing interests.

## Ethical considerations

Ethical issues (including plagiarism, data fabrication, double publication) have been completely observed by the author.

## Funding/Support

None.
